# Bioactive Lipids as Mediators of the Beneficial Action(s) of Mesenchymal Stem Cells in COVID-19

**DOI:** 10.14336/AD.2020.0521

**Published:** 2020-07-23

**Authors:** Undurti N Das

**Affiliations:** ^1^UND Life Sciences, Battle Ground, WA 98604, USA; ^2^BioScience Research Centre and Department of Medicine, Gayatri Vidya Parishad Medical College and Hospital, Visakhapatnam-530048, India.

**Keywords:** COVID-19, immune check point inhibitory therapy, cytokines, inflammation, bioactive lipids, mesenchymal stem cells

## Abstract

It is proposed that the beneficial action of mesenchymal stem cells (MSCs) in COVID-19 and other inflammatory diseases could be attributed to their ability to secrete bioactive lipids (BALs) such as prostaglandin E2 (PGE2) and lipoxin A4 (LXA4) and other similar BALs. This implies that MSCs that have limited or low capacity to secrete BALs may be unable to bring about their beneficial actions. This proposal implies that pretreatment of MSCs with BALs enhance their physiological action or improve their (MSCs) anti-inflammatory and disease resolution capacity to a significant degree. Thus, the beneficial action of MSCs reported in the management of COVID-19 could be attributed to their ability to secrete BALs, especially PGE2 and LXA4. Since PGE2, LXA4 and their precursors AA (arachidonic acid), dihomo-gamma-linolenic acid (DGLA) and gamma-linolenic acid (GLA) inhibit the production of pro-inflammatory IL-6 and TNF-α, they could be employed to treat cytokine storm seen in COVID-19, immune check point inhibitory (ICI) therapy, sepsis and ARDS (acute respiratory disease). This is further supported by the observation that GLA, DGLA and AA inactivate enveloped viruses including COVID-19. Thus, infusions of appropriate amounts of GLA, DGLA, AA, PGE2 and LXA4 are of significant therapeutic benefit in COVID-19, ICI therapy and other inflammatory conditions including but not limited to sepsis. AA is the precursor of both PGE2 and LXA4 suggesting that AA is most suited for such preventive and therapeutic approach.

The pandemic of COVID-19 is caused by the novel coronavirus that belongs to the severe acute respiratory syndrome coronavirus (SARS-CoV) phylogenetically and can spread from person to person very easily. COVID-19 manifests itself as fever, severe respiratory illness and pneumonia. COVID-19 is caused by SARS-CoV-2 and it belongs to the SARS-CoV and Middle East respiratory syndrome coronavirus (MERS-CoV)- all of these viruses belong to the beta coronavirus genus. Like all the coronaviruses, SARS-CoV-2 is also spherical and the spike glycoprotein (S) on the COVID-19 surface can bind to the ACE2 receptor for cell entry. Despite similarities in sequence and structure between the spikes of the SARS-CoV and SARS-CoV-2 viruses, antibodies against the 2002 SARS virus could not bind to the COVID-19 spike protein, suggesting that there are significant structural differences between the two viruses [1]. These results suggest that potential treatment strategies need to be more specific to COVID-19.

Previously I suggested that bioactive lipids such as gamma-linolenic acid (GLA), dihomo-GLA (DGLA), the precursor of prostaglandin E1 (PGE1); arachidonic acid (AA), the precursor of both PGE2 and lipoxin A4 (LXA4); eicosapentaenoic acid (EPA), the precursor of PGE3 and E series resolvins; and docosahexaenoic acid (DHA), the precursor of D series resolvins, protectins and maresins; are capable of inactivating enveloped viruses including SARS-CoV-2 [2, 3] and hence, are of significant use in the treatment of COVID-19. In a recent study, Leng et al [4] reported that mesenchymal stem cells (MSCs) transplantation in those who were positive for SARS-CoV-2 can induce remarkable improvement with a significant drop in systemic inflammation. This raises the question as to what potential relationship exists between the beneficial action of MSCs and the role of bioactive lipids in COVID-19.

## Anti-inflammatory action of MSCs

Mesenchymal stromal cells (MSCs) have been explored as potential therapeutic option for inflammatory conditions [5, 6). MSCs are multipotent stromal cells and are present in many tissues and differentiate into several different cell types to bring about their beneficial actions. Exogenously administered MSCs are capable of migrating to damaged tissue sites and participate in tissue repair. MSCs are capable of communicating with the inflammatory microenvironment and depending on the type and intensity of inflammation, they suppress or enhance immune response. Studies revealed that administration of MSCs and hemopoietic stem cells suppress the production of interleukin (IL)-6, tumor necrosis factor-alpha (TNF-α), transforming growth factor-beta (TGF-β), and IL-10, and inhibit the expression of nuclear factor-kappaB (NF-κB), toll-like receptor-2 (TLR-2), matrix metalloproteinase-3 (MMP-3) and cartilage oligomeric matrix protein-1 (COMP-1) genes in a rat model of rheumatoid arthritis [6].

It is acknowledged that defective clearance of apoptotic cells (ACs) may play a role in the persistence of inflammation in many diseases especially in the pathogenesis of lupus in which use of MSCs showed promise. When human umbilical cord (UC) MSCs were co-cultured with ACs, they engulfed (human UC MSCs) ACs, showed (i) suppression of CD4^+^ T cell proliferation, (ii) increased expression of COX-2 (cyclo-oxygenase-2), (iii) produced higher amounts of PGE2 that inhibited T cell response and (iv) NF-kB signaling pathway mediated the activation of COX-2/PGE2 compared to MSCs alone. It is noteworthy that in patients with lupus, the plasma PGEM (PGE metabolites) levels were increased significantly in those with reduced apoptotic mononuclear cells in peripheral blood after MSC transplantation [7]. These results suggest that MSCs induce immunosuppression by enhancing PGE2 production and defective clearance of apoptotic cells is due to enhanced metabolism of PGE2 (ACs) leading to a decrease in their (PGE2) levels. These results imply that MSCs contribute to immunosuppression by elaborating PGE2 and methods designed to enhance PGE2 levels may be of benefit in lupus and other similar inflammatory conditions.

In this context, it is interesting to note that human mesenchymal stromal/stem cells (hMSCs) that are used in cell therapy to treat immunological disorders, and their extracellular vesicles (hMSC-EVs) have immunomodulatory function and induce resolution of inflammation. It was reported that hMSCs supplemented with AA, EPA and DHA were incorporated into the main membrane phospholipid classes predominantly into phosphatidylcholine. Even hMSCs-EVs also showed similar PUFA modifications. The most significant finding of this study is the observation that supplemented AA, EPA and DHA resulted in enhanced production of PGE2, resolvin E2 and resolvin D6. It is significant to know that the production of PGE2 (from AA), resolvin E2 (from EPA) and resolvin D6 (from DHA) is enhanced in parallel to the proinflammatory stimulus: the higher the proinflammatory stimulus the higher the production of PGE2, resolvin E2 and resolvin D6 [8]. This implies that the ability hMSCs and hMSC-EVs response to proinflammatory stimulus parallels the strength of the stimulus. These results can be interpreted to mean that hMSCs and hMSC-EVs need to contain sufficient amounts of AA, EPA and DHA to be able to produce enough of PGE2, resolvin E2 and resolvin D6 respectively to respond in an adequate fashion to the proinflammatory stimulus. It is noteworthy that these MSCs and MSC-EVs act on mature human regulatory macrophages (Mregs) and downregulated their production of IL-1β, IL-2, IL-5, TNF-α, IL-23 and IL-22 (IL-22 and IL-23 inhibit pathogenic Th17 cells and thus, suppress inflammation), enhanced their PGE2, 15-HETE, 18-HEPE, 17-HDHA, and 14S-HDHA production (some of which act as precursors to resolvins, protectins and maresins) that enhance the anti-inflammatory phenotype of Mregs and amplifies their proresolving properties [9]. These results led to the proposal that enhanced PGE2 production is necessary for proresolving lipid mediators class switching such that pro-inflammatory eicosanoids production is suppressed and anti-inflammatory eicosanoids are produced. Interestingly, RvD1, RvD2, and RvE1 are known to reduce IL-23 production in asthma, microbial peritonitis, and allergic airway mouse models [10-12]. These data suggest that both human MSC and MSC-EVs may potentiate tolerance-promoting proresolving phenotype of human Mregs. These results are sponsored by several other studies that revealed that PGE2 has a central regulatory role in mediating the anti-inflammatory actions of MSCs [13-17]. It is interesting to note that the ability of MSCs to alter the functional status of macrophages (M1 to M2 and *vice versa*) is dependent on their ability to produce PGE2 [17]. MSCs have the property of promoting macrophage differentiation, enhance their respiratory burst activity and potentiate microbicidal responses in naïve macrophages (Mφ). MSc-M1 co-culture attenuated inflammatory M1 macrophages and shifts them to activated M2 state, whereas MSC-M2 co-cultures led to generation of M1 status. Studies performed with Mφ/MSC, M1/MSC and M2/MSC co-cultures revealed changes in Glucose transporter1 (GLUT1 expression/glucose uptake, IDO1 (indoleamine 2, 3-dioxygenase) protein/activity, SIRTUIN1 and alterations in AMPK and mTOR activity occurs reflecting MSC-instructed metabolic shifts. COX-2 knockdown abolished this ability of MSCs to attenuate M1 macrophages and inducing metabolic shifts in polarized macrophages indicating a key role for MSC-secreted PGE2 in their (MSCs) ability to manipulate macrophage metabolic status and plasticity [17]. Thus, PGE2 production by MSCs seems to be crucial for macrophage differentiation and plasticity by re-educating macrophages by manipulating their metabolic programs.

**Figure 1.**
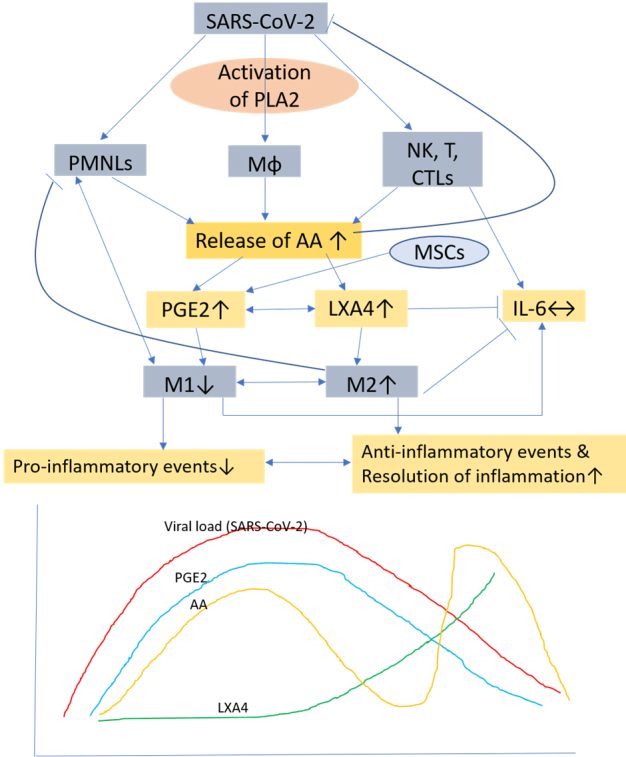
**Scheme showing potential relationship among AA, PGE2, LXA4 and viral load in a COVID-19 patient who recovers**. AA is released from the cell membrane in two phases, first phase is used for PGE2 synthesis, whereas the second phase is meant for LXA4 synthesis. Once PGE2 concentration reaches its peak, LXA4 synthesis is triggered that induces resolution of inflammation. AA release is triggered by SARS-CoV-2 and other infections.

Adipose tissue-derived MSCs (ASCs) inhibit secretion of IL-1β and IL-18 but show increased PGE2 production that, in turn, inhibited inflammasome complex formation and M1 macrophage population and increased regulatory T cells in the DSS-induced chronic colitis model suggesting suppression of the inflammatory response [18, 19]. In addition, human MSCs can reciprocally regulate human Th17 and Th1 responses in a PGE2-dependent and myeloid cell-mediated manner implying a potential therapeutic role in immune-mediated diseases [20]. MSCs have been shown to modulate CD4 T lymphocyte population by secreting transforming growth factor β1 (TGF-β1) and PGE2 that inhibit T cell proliferation, polarize naïve CD4 towards a regulatory T cell (Treg) phenotype, shifting the cytokine profile from a T helper cell type 1 (Th1)—in which high levels of interferon-γ (IFN-γ) and TNF-α are secreted—to a Th2 milieu [22] and inhibit the cytotoxic activity of CD8 cytotoxic T cells, natural killer cells (NK), and interfere with B cell maturation and antibody production [21-28].

**Figure 2.**
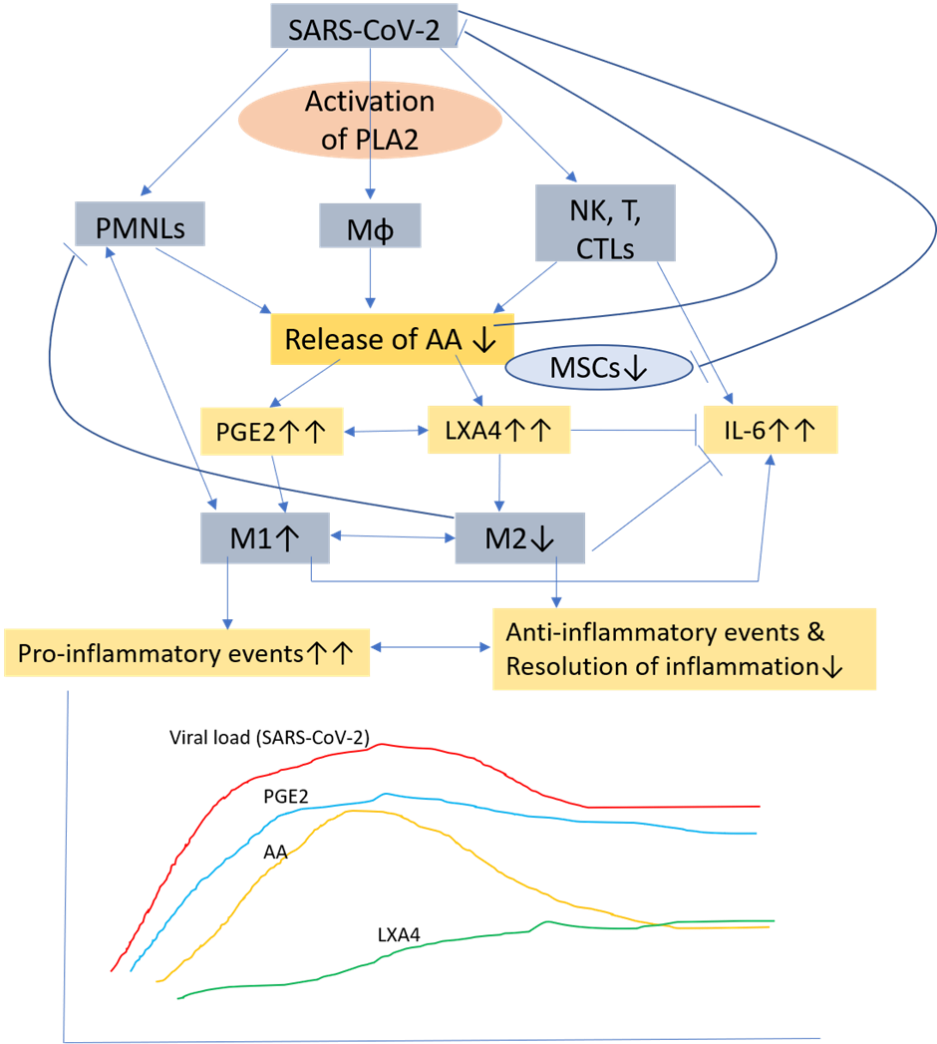
**Scheme showing potential relationship among AA, PGE2, LXA4 and viral load in a COVID-19 patient who succumbs to the disease**. Note the absence of biphasic nature of AA release and failure of PGE2 to reach its peak and as a result deficiency in LXA4 synthesis occurs that results in failure of resolution of inflammation (compare with Fig 1). DGLA, EPA and DHA (not shown in the figure) may have actions/functions like AA.

## Interaction between PGE2 and LXA4

In general, PGE2 is regarded as a pro-inflammatory molecule participating in the initiation of inflammation. However, PGE2 also possesses anti-inflammatory and immunosuppressive properties [21, 29] especially by its ability to alter macrophage polarization by MSCs (30-32). This dual action of PGE2 seems to be due to the differing activities of PGE2 receptors (EPs). At low PGE2 concentrations, the high-affinity EP4 increased IL-23 production [33]. In contrast, high PGE2 amounts act on EP2 to downregulate the IL-23 release [34, 35]. Thus, MSCs and MSC-EVs can suppress IL-23 production by producing high levels of PGE2 and IL-10 [13-17, 36]. In addition, PGE2 can induce lipid mediator class switching (from pro-inflammatory to anti-inflammatory) in neutrophils by modulating 5- and 15-lipoxygenase expression that results in inhibition of pro-inflammatory leukotriene B4 (LTB4) and increased production of LXA4 (a potent anti-inflammatory molecule) that are needed to remove neutrophils from the site of inflammation and to restore homeostasis. This process is dependent on macrophage production of PGE2 that drives neutrophil removal by promoting reverse migration of neutrophils [37]. This crosstalk among macrophages and neutrophils and PGE2 and LXA4 is crucial to dampen inflammation. In this context, it is noteworthy that PGE1, derived from DGLA, is an anti-inflammatory, vasodilator and platelet anti-aggregator whose actions are like those of LXA4. It is not known whether PGE1 also can regulate LXA4 formation like PGE2. It is anticipated that the interaction between PGE1 and LXA4 is akin to that of PGE2 and LXA4.

## PGE2 enhances tissue regeneration

Thus, the anti-inflammatory action of PGE2 seem to depend on its ability to enhance lipoxin A4 (LXA4) formation [38], a potent anti-inflammatory molecule. This implies that once the inflammation reaches a peak in which PGE2 plays a role, the metabolism of AA, the precursor of both PGE2 and LXA4, need to be redirected to form LXA4 instead of PGE2 (see Fig. 1 and 2). This switching of AA metabolism from PGE2 to LXA4 is a crucial event in the resolution of inflammation [39, 40]. It is interesting to know that 15-PGDH-(15-prostaglandin dehydrogenase, a prostaglandin degrading enzyme) deficient mice not only have a twofold increase in PGE2 levels in several tissues especially in the bone marrow, colon, and liver but also showed increased fitness of these tissues with enhanced hematopoietic capacity. These 15-PGDH deficient animals showed rapid liver regeneration after partial hepatectomy and showed accelerated recovery of neutrophils, platelets, and erythrocytes [41]. These results are supported by the observation that PGE2 regulates haematopoietic stem cell homeostasis, promotes wound healing and tissue regeneration and modulates stem cell trafficking, events that ultimately promote hematopoiesis [42-45]. Furthermore, PGE2, LXA4 and other bioactive lipids such as AA, EPA and DHA and their other antiinflammatory metabolites resolvins, protectins and maresins are all potent inhibitors of IL-6, TNF-α and other pro-inflammatory cytokines [39, 40]. Based on these evidences, it is suggested that administration of AA/PGE2/LXA4 and other bioactive lipids to those with SARS-CoV-2 (COVID-19) infection not only inactivate the virus [2, 3, 46] but may also abrogate fulminant activation of coagulation, microvascular thrombosis and consumption of coagulation factors as evidenced by thrombocytopenia, prolongation of the PT/INR, PTT, elevation of D-dimer, and decreased fibrinogen levels, seen in those with severe COVID-19 that are believed to be due to cytokine storm induced by the infection.

## LXA4 mediates MSCs resolution of lung inflammation

Bone marrow derived MSCs reduce the severity of acute lung injury in animal models and in an ex vivo perfused human lung model by secreting proresolving mediator LXA4. Both human alveolar epithelial type II cells and MSCs express biosynthetic enzymes and receptors for LXA4. Human MSCs are known to secrete LXA4. Blocking the LXA4 receptor was found to significantly reverse the protective effect of MSCs on both survival and the accumulation of pulmonary edema. LXA4 alone has been shown to improve survival and attenuate LPS-induced acute lung injury in mice by significantly decreasing the production of TNF-α and MIP-2 in bronchoalveolar lavage fluid. These studies suggest that human MSCs promote the resolution of lung injury by secreting LXA4 [47, 48].

MSCs treatment is effective in diabetic nephropathy, an inflammatory condition, by protecting renal function and preventing fibrosis. Studies performed in diabetic nephropathy model induced by streptozotocin in SD rats, showed that MSCs treatment significantly increased LXA4 and ALX/FPR2, the receptor of LXA4, expression in renal tissue of these animals. Intraperitoneal administration of LXA4 inhibited renal fibrosis by suppressing serum TNF-α, IL-6, IL-8, and IFN-γ in diabetic nephropathy rats. All these protective effects induced by MSCs or LXA4 could be abolished by ALX/FRP2 blocking, suggesting that MSCs prevent diabetic nephropathy via the LXA4-ALX/FPR2 axis, which inhibited pro-inflammatory cytokines [49]. Thus, MSCs bring about their beneficial actions by elaborating anti-inflammatory LXA4 and other bioactive lipids. This is further supported by our studies that showed that streptozotocin-induced type 1 and type 2 diabetes mellitus in experimental animals can be prevented by AA, LXA4 and other bioactive lipids by suppressing the production of pro-inflammatory cytokines [50-56].

**Figure 3.**
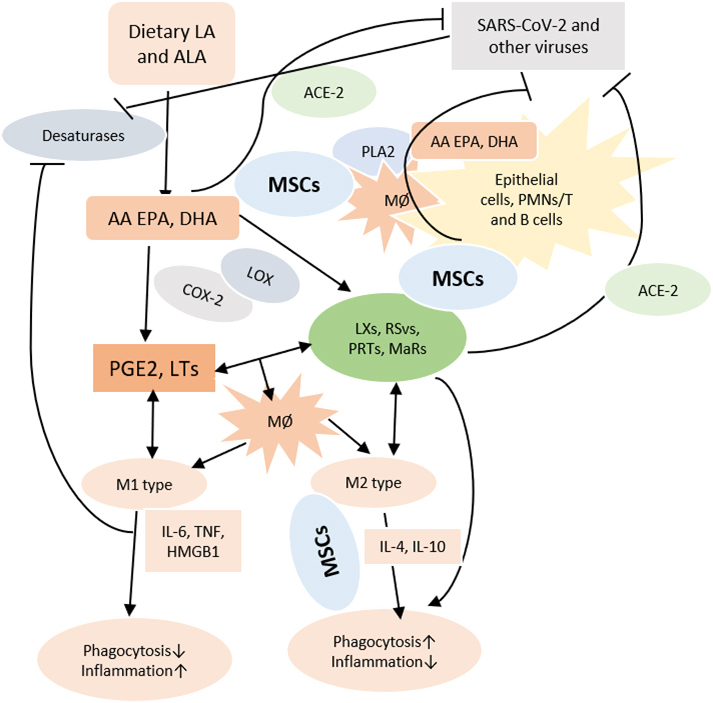
**Scheme showing possible role of MSCs and bioactive lipids in COVID-19**. Dietary LA and ALA are converted to AA and EPA and DHA by desaturases. Viruses block the activities of desaturases. PGE2 and LTs derived from AA/EPA/DHA facilitate M1 generation and enhance inflammation. Anti-inflammatory PGE1/PGE2, lipoxins, resolvins, protectins and maresins derived from DGLA, AA, EPA and DHA facilitate M2 generation and suppress inflammation. PLA2 activated by SARS-CoV-2 and other viruses induce the release of DGLA/AA/EPA/DHA that inactivate enveloped viruses by themselves and through their products and regulate inflammatory process. MSCs and other stem cells contain DGLA/AA/EPA/DHA and release PGE2 and LXA4 and other bioactive lipids to resolve inflammation. DGLA/AA/EPA/DHA by their ability to alter cell membrane composition can regulate ACE2 expression. ACE2 is needed for SARS-CoV-2 entry into the cells.

## Targeted inhibition of PGE2 enhances anti-viral immunity

Aspirin, an age-old anti-inflammatory drug, is a potent inhibitor of PG synthesis (57). Aspirin was popular during the 1918 Spanish Influenza virus pandemic though its beneficial actions remain controversial. It is known that in influenza A virus (IAV) infection, PGE2 synthesis is upregulated that, in turn, inhibited type I interferon (IFN) production and apoptosis of macrophages that increased virus replication. PGE2 not only inhibited innate immunity but also suppressed both antigen presentation and T cell mediated immunity. Coulombe et al [58] showed that targeted PGE2 suppression via genetic ablation of microsomal prostaglandin E-synthase 1 (mPGES-1) or by the pharmacological inhibition of PGE2 receptors EP2 and EP4 significantly improved survival against lethal IAV infection. In contrast, PGE2 administration enhanced mortality. This led to the suggestion that strategies designed to specifically inhibit the mPGES-1-PGE2 pathway could be of significant benefit in the treatment of IAV. Though this idea is appealing, it may have another interesting interpretation. As discussed above, it is possible that when PGE2 synthesis is blocked in a specific fashion, there could occur a concomitant increase in LXA4 synthesis since both PGE2 and LXA4 are derived from the same precursor namely AA. Hence, it is suggested that inhibition of mPGES-1-PGE2 pathway leads to an increase in LXA4 formation and thus, is of benefit in the treatment of IAV and COVID-19.

## Conclusions and therapeutic implications

Based on the preceding discussion, it is proposed that MSCs (and other types of stem cells) transplantation in those who were positive for SARS-CoV-2 showed remarkable improvement with a significant drop in systemic inflammation [4] could be attributed to the secretion of PGE2 and LXA4 (predominantly due to LXA4 formation with an initial increase in PGE2 synthesis that would trigger LXA4 synthesis once the concentrations of PGE2 reach a threshold) (see Fig. 1 and 3) and other bioactive lipids by the infused MSCs. This could be verified in future studies by measuring plasma levels of various bioactive lipids before and after MSCs and correlating the same to the changes in the clinical picture. It is suggested that treatment of MSCs with bioactive lipids may further enhance their function and shorten the recovery period by enhancing their ability to suppress pro-inflammatory cytokine production, NK cell proliferation and activity [9, 10,59]. It will be interesting to study the plasma, urine and alveolar fluid concentrations of PGE2, LXA4 and other bioactive lipids in patients with COVID-19 and correlate the same to the clinical picture and the changes in the profile of various cytokines. It is anticipated that in those with COVID-19 and other similar inflammatory diseases such as sepsis and ARDS (acute respiratory distress syndrome) there would occur an initial increase in PGE2 levels followed by a gradual and smooth transition in the formation and increase in the concentrations of LXA4 as the patients show improvement in their clinical condition. In those who are likely to succumb to the disease (COVID-19, ARDS and sepsis), there will be a sustained and persistance increase in PGE2 levels without the anticipated transition in LXA4 formation that would result in failure of resolution of inflammation and hence are likely to succumb to the disease. It is likely that the changes in PGE2 and LXA4 levels will reflect in similar changes in the concentrations of pro- and anti-inflammatory cytokines-initial increase in IL-6 and TNF-α and other pro-inflammatory cytokines and those who recover will show a gradual and sustained increase in anti-inflammatory cytokines whereas those who succumb this transition from pro- to anti-inflammatory status would not occur.

Based on these arguments, it is proposed that after understanding the dynamics of PGE2 and LXA4, it is possible administer PGE2 and LXA4 one after the other to mimic the natural process of recovery and initiate and induce resolution of inflammation in the lungs and other tissues and improve prognosis of COVID-19, ARDS and sepsis. It is suggested that perhaps, infusion of LXA4 or its analogues, especially PGDH-resistant lipoxin analogues could be effective. Though it is tempting to speculate that administration of AA, the precursor of both PGE2 and LXA4, may result in the formation of PGE2 and LXA4 as per the necessity and lead to resolution of inflammation needs to be verified in future studies. Another possibility is to study the potential use of PGE1 (derived from DGLA) that is similar in structure and action to PGE2 except that it is a platelet anti-aggregator (in addition to its ability to suppress IL-6 and TNF-α synthesis, anti-inflammatory and vasodilator actions) whereas PGE2 is a platelet aggregator in COVID-19, ARDS and sepsis. It is anticipated that PGE1 may be more beneficial compared to PGE2. It remains to be seen whether AA alone, or a combination of PGE2-LXA4, PGE1-LXA4 and AA-LXA4 are best suited in the management of COVID-19, ARDS and sepsis.

Thus, the close cellular and molecular mechanisms of the interaction among MSCs, various participants in inflammation and bioactive lipids needs further study.
